# Antioxidative properties analysis of gastrointestinal lactic acid bacteria in Hainan black goat and its effect on the aerobic stability of total mixed ration

**DOI:** 10.3389/fmicb.2022.974925

**Published:** 2022-08-30

**Authors:** Tianshu Yang, Jinsong Yang, Kai Tang, Wenbo Zhi, Rong Chen, Haisheng Tan

**Affiliations:** ^1^College of Food Science and Engineering, Hainan University, Haikou, China; ^2^College of Materials Science and Engineering, Hainan University, Haikou, China

**Keywords:** Hainan black goat, lactic acid bacteria, antioxidant, total mixed ration, aerobic stability

## Abstract

In this study, lactic acid bacteria strains (HCS-01, HCS-05, HCS-07, HCW-08, and HCW-09) derived from the gastrointestinal tract of Hainan black goat were evaluated for their antioxidant capacity *in vitro*, and the lactic acid bacteria with strong antioxidant capacity were screened for application to improve the aerobic stability of total mixed ration (TMR). The results showed that all the tested lactic acid bacteria had a certain tolerance to hydrogen peroxide. By comprehensively comparing the scavenging abilities of fermentation supernatants, whole cell bacterial suspensions and cell contents of five lactic acid bacteria strains to 2,2-diphenyl-1-picrylhydrazine (DPPH), hydroxyl radicals and superoxide anions, and their antioxidant enzyme activity, it was found that *Lactobacillus fermentum* HCS-05 and *Lactobacillus plantarum* HCW-08 have the strongest comprehensive antioxidant capacity, and their scavenging capacity for various free radicals has reached more than 60%. Using strains HCS-05, HCW-08 and laboratory-preserved *Lactobacillus plantarum* HDX1 fermented TMR, the fermentation quality and aerobic stability of the feed after 60 days of fermentation were significantly higher than those of the blank treatment group. The effect of mixed strains HCS-05 and HCS-08 for TMR fermentation was the best (*P* < 0.05). At the same time, the fermentation effect of *Lactobacillus plantarum* HDX1 on TMR was significantly lower than that of the selected lactic acid bacteria from the gastrointestinal tract of Hainan black goats (*P* < 0.05). The results show that the test strain can significantly improve the aerobic stability of the fermented feeds.

## Introduction

Lactic acid bacteria is a natural antioxidant, and its antioxidant capacity has been proven from multiple perspectives, mainly in scavenging free radicals, regulating antioxidant-related enzymes and the dynamic balance of intestinal microflora (Kullisaar et al., 2002; Zhang et al., 2017; Dowarah et al., 2018). The antioxidant activity of lactic acid bacteria can alleviate the oxidative stress response in the body caused by the imbalance between reactive oxygen species or free radicals and antioxidant defense in the body, and inhibit the aging lesions caused by oxidative damage in the body (Zhang et al., 2013; Chooruk et al., 2017). With the development and application of the antioxidant activity of lactic acid bacteria, the screening of lactic acid bacteria strains with strong antioxidant capacity or specificity has also become particularly important (Ahotupa et al., 1996). Hainan black goat is a ruminant animal with a wide variety of microorganisms in its gastrointestinal tract and rich lactic acid bacteria resources. The lactic acid bacteria screened from the gastrointestinal tract of animals usually have better acid-producing and acid-resisting ability due to their original growth environment, and the ability to adapt to the gastrointestinal tract environment is also stronger.

Total mixed ration (TMR) because of the high water content and the large proportion of silage raw materials, aerobic microorganisms are more active, and the quality stability is usually poor, so it is not suitable for storage (Li et al., 2021). In the process of making and taking silage, there is often the problem of secondary fermentation, which will cause problems and losses in its economic benefits and popularization and application. Improving the aerobic stability of feed has been a point of concern in feed research. Since TMR fermented feed contains more abundant nutrients, the research on its aerobic stability has become particularly important. In order to overcome this problem, some scholars have begun to conduct in-depth research on fermenting TMR to FTMR (Fermented Total Mixed Ration) for long-term storage. Kim et al. (2012) suggested that FTMR had better *in vitro* rumination characteristics than TMR, and the growth performance and blood characteristics of Hanwoo cattle fed FTMR were also better than TMR and controls; Panyawoot et al. (2022) found that FTMR treated with *Lactobacillus casei* TH14 had higher apparent nutrient digestibility compared to unfermented TMR, benefiting goat growth without negative effects. This indicates that FTMR is more beneficial to the growth and development of ruminants than TMR and that lactic acid bacteria play a significant positive role in TMR fermentation. Using lactic acid bacteria derived from animals to make special micro-ecological preparations and applying them to the fermentation of feeds eaten by animals may increase the number of beneficial microorganisms in their gastrointestinal flora and become the dominant flora, and at the same time, it also has higher security (Yuan et al., 2015).

In this study, in order to improve the stability of TMR and prolong the storage time, so as to solve the problem of seasonal feed shortage and indirectly promote animal growth and development, the antioxidant properties of lactic acid bacteria derived from the gastrointestinal tract of Hainan black goat were analyzed and its effect on TMR fermentation was studied. This study provides strain resources and practical guidance for optimizing TMR feed fermentation technology and improving the large-scale breeding technology of Hainan black goat.

## Materials and methods

### Samples and strains

Lactic acid bacteria strains: *Lactobacillus plantarum* HCS-01, HCS-07, HCW-08, *Lactobacillus fermentum* HCS-05, *Lactobacillus salivarius* HCW-09 stored in the laboratory were all derived from the gastrointestinal tract of Hainan black goats; Reyan No. 4 king grass; Refined feed.

### Reagents and instruments

#### Main reagents

2,2-diphenyl-1-picrylhydrazine (DPPH), pyrogallol, ferric chloride, *o*-phenoline, tris (Tris): Shanghai McLean Biochemical Technology Co., Ltd.; Antibiotic test strips: Beekman Bio; SOD kit, CAT kit, GSH-PX kit: Shanghai Yuanye Biotechnology Co., Ltd.

#### Main instruments

SW-CJ-17-D ultra-clean workbench: Shanghai Hetai Instrument Co., Ltd.; SPX-250B-Z constant temperature biochemical incubator, D3024 micro high-speed centrifuge: Guilin Instrument Equipment Co., Ltd.; Hettich32R high-speed refrigerated centrifuge: German ZENTRIFMGEN; Synergy LX microplate reader: American BIOTEK.

### Methods

#### Preparation of samples of each component of lactic acid bacteria

The lactic acid bacteria strains to be tested stored in the ultra-low temperature refrigerator at −80°C were activated in MRS liquid medium. MRS liquid medium was inoculated with 2% of the test bacterial solution, and incubated at 37°C under constant temperature for 24 h. The fermentation broth was centrifuged at 10,000 × g (D3024 micro high-speed centrifuge) for 15 min, the supernatant was collected, and filtered with a 0.22 μm microporous membrane filter to obtain the fermentation supernatant; the bacteria were collected, washed with PBS buffer and resuspended to adjust the number of bacteria to 10^9^ CFU/mL to obtain a complete cell bacterial suspension; a part of the bacterial suspension was sonicated in an ice bath, and the supernatant was collected after centrifugation to obtain a cell-free extract, the cell content (Dobrowolski et al., 2012).

#### Tolerance of lactic acid bacteria to hydrogen peroxide

The test bacterial solution was inoculated in MRS medium supplemented with H_2_O_2_ (concentrations of 0.0, 1.0, 2.0, and 3.0 mmol/L) according to 2% of the volume fraction, and cultured at 37°C for 24 h., 12 h and 24 h sampling and measuring its Absorbance (OD = 600) (Amanatidou et al., 2001b).

#### Determination of free radical scavenging ability of lactic acid bacteria

##### 2,2-Diphenyl-1-picrylhydrazine radical scavenging activity assay

The DPPH radical scavenging activity of lactic acid bacteria was determined by referring to the method in the literature (Rubio et al., 2014), and the DPPH radical scavenging rate was calculated according to the formula:


(1) DPPHradicalscavengingrate(%)

$$=\left[1-\left({\text{A}}_{1}-{\text{A}}_{0}\right)\,/\,{\text{A}}_{2}\right]\times 100$$

In the formula: A_1_ is the absorbance of the sample group; A_0_ is the absorbance of the blank group; A_2_ is the absorbance of the control group.

##### Hydroxyl radical scavenging activity assay

The hydroxyl radical scavenging activity of lactic acid bacteria was determined with reference to the method in the literature (Kuda et al., 2014), and the Hydroxyl radical scavenging rate was calculated according to the formula:


(2) Hydroxylradicalscavengingrate(%)

$$=\left[\left({\text{A}}_{1}-A_{3}\right)\,/\,\left({\text{A}}_{2}-A_{3}\right)\right]\times 100$$

In the formula: A_1_ is the absorbance of the sample group; A_2_ is the absorbance of the control group; A_3_ is the absorbance of the blank group.

##### Superoxide anion scavenging activity assay

The superoxide anion scavenging activity of lactic acid bacteria was determined with reference to the method of the literature (Chen et al., 2015), and the hydroxyl radical scavenging rate was calculated according to the formula:


(3) Superoxideanionscavengingrate(%)

$$=\left[1-{\text{A}}_{1,/}\,{\text{A}}_{2}\right]\times 100$$

In the formula: A_1_ is the absorbance of the sample group; A_2_ is the absorbance of the blank group.

#### Antioxidant enzyme activity of lactic acid bacteria

The activities of catalase (CAT), superoxide dismutase (SOD), and glutathione peroxidase (GSH-PX) in the prepared samples of lactic acid bacteria were measured by using CAT, SOD, and GSH-PX, reagent test kit (Shanghai Yuanye Biotechnology Co., Ltd.).

#### The effect of lactic acid bacteria fermentation on the aerobic stability of total mixed ration

##### Fermentation total mixed ration modulation

According to the above identification results of lactic acid bacteria and the analysis of probiotic characteristics, the most suitable strains HCS-05 and HCW-08 for silage fermentation were selected as the biological starter for this TMR silage fermentation test, and the laboratory-preserved strain HDX1 was used as the control starter. The king grass crushed by the pulverizer and the concentrate were mixed at a ratio of 4:6 to form TMR (Somashekaraiah et al., 2019), which was put into silage bags (1,000 g per bag). The prepared TMR were divided into four groups, and the treatments of each group are shown in Table 1. The treated TMR in each group were evacuated and sealed, then placed at room temperature and stored in the dark for 60 days.

**TABLE 1:** Treatment method of total mixed ration (TMR).

No.	Approach	Addition amount/kg	Amount/Pack	The amount of lactic acid bacteria added
I	TMR	1.0	18	–
II	TMR + HCS-05	1.0	18	0.1%
III	TMR + HCS-08	1.0	18	0.1%
VI	TMR + HCS-05 + HCS-08	1.0	18	0.1%
V	TMR + HDX1	1.0	18	0.1%

##### Determination of total mixed ration nutrients and fermentation quality

Determine the dry matter content (DM), crude protein (CP), and water-soluble carbohydrate (WSC) content of each group of samples after 60 days of fermentation, determination of pH, organic acids and ammonia nitrogen in silage (Nishino et al., 2010).

Referring to the method of Yang et al. (2012), after 60 days of fermentation of TMR silage, samples were taken from the opening of the package, the pH was measured and the microbial growth was analyzed.

##### Aerobic stability analysis of fermented total mixed ration

After the 60-day fermentation, the silage bags treated in each group were opened and mixed, and then placed openly. Samples were taken on the 0th, 3rd, 6th, 9th, and 12th days, and the pH value and microbial growth were measured. Changes in feed internal temperature during aerobic exposure were detected and recorded using a thermometer and compared to room temperature (Gomes et al., 2021).

### Data analysis

Using SPSS 22 and GraphPad Prism 9 for data analysis, using Origin 9.0 for graphing.

## Results and discussion

### Analysis of tolerance of lactic acid bacteria to hydrogen peroxide

The antioxidant capacity of lactic acid bacteria is a part of all the functional properties of lactic acid bacteria that has always been valued (Rizzello et al., 2017). Hydrogen peroxide is an oxidant with high diffusivity and long acting time. The ability to tolerate hydrogen peroxide is an indicator to evaluate the antioxidant capacity of lactic acid bacteria (Lee et al., 2005).

**FIGURE 1:**
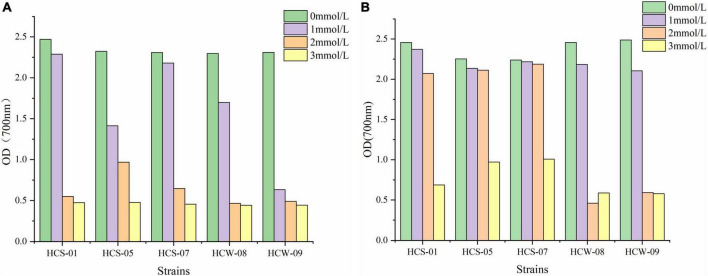
Growth of strains at different concentrations of H_2_O_2_. **(A)** 12 h, **(B)** 24 h.

The growth of lactic acid bacteria in liquid medium supplemented with different concentrations of H_2_O_2_ is shown in Figure 1. The growth of 12 h of culture is shown in Figure 1A. The growth of each strain was significantly different under different concentrations of H_2_O_2_. With the increase of H_2_O_2_ concentration, the growth ability of each strain decreased overall, but the degree of decline was different. When the H_2_O_2_ concentration was 0 and 1 mmol/L, most of the strains grew well, and the OD_600_ was above 1.4, and the growth of HCW-09 was weak; when the H_2_O_2_ concentration was 2 and 3 mmol/L, all strains grew The ability of the strains decreased rapidly, and the OD_600_ were all below 1; when the strains were cultured for 24 h, their growth was basically the same as the change trend with H_2_O_2_ concentration at 12 h (Figure 1B). All strains can tolerate the long-term action of H_2_O_2_, and HCS-05 and HCS-07 were more tolerant to hydrogen peroxide.

### Analysis of the free radical scavenging activity of lactic acid bacteria

#### Analysis of 2,2-diphenyl-1-picrylhydrazine radical scavenging activity

**FIGURE 2:**
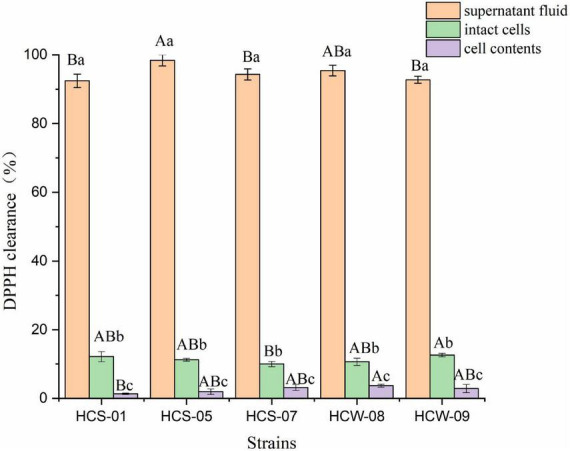
2,2-Diphenyl-1-picrylhydrazine (DPPH) free radical scavenging ability of lactic acid bacteria. Different lowercase letters indicate significant differences among different components of the same strain (*P* < 0.05); different capital letters indicate significant differences among the same components of different strains (*P* < 0.05).

2,2-Diphenyl-1-picrylhydrazine free radicals are an important indicator for evaluating the *in vitro* antioxidant capacity of substances, and the antioxidant capacity of lactic acid bacteria is positively correlated with the ability to scavenge DPPH free radicals (Liochev, 2013). The DPPH free radical scavenging ability of different components of lactic acid bacteria is shown in Figure 2. The three components of the five lactic acid bacteria all showed a certain DPPH free radical scavenging ability, and the DPPH free radical scavenging rate of the fermentation supernatant was significantly higher than the other two Component (*P* < 0.05), the scavenging rate of DPPH free radicals in the fermentation supernatant of the five lactic acid bacteria were all above 90%. Among them, the highest scavenging rate of HCS-05 was 98.39%, which may be due to the fact that the fermentation supernatant of lactic acid bacteria contains a large number of active substances such as organic acids with reducing properties produced by lactic acid bacteria during the fermentation process (Zhang et al., 2018); the DPPH free radical scavenging rate of the complete cell suspension of each strain was lower, but significantly higher than that of the cell content (*P* < 0.05), among which HCW-09 scavenged The highest rate is 12.59%, and the lowest is 9.97% for HCS-07, which may be because *Lactobacillus* exopolysaccharides with a certain hydrogen-donating ability can be produced on the surface of intact bacterial cells, neutralizing DPPH free radicals through hydrogen atom transfer or electron transfer (Li et al., 2012). The cell contents of lactic acid bacteria showed a lower DPPH free radical scavenging ability, and the scavenging rate ranged from 1.33 to 3.71%.

#### Analysis of hydroxyl radical scavenging activity

**FIGURE 3:**
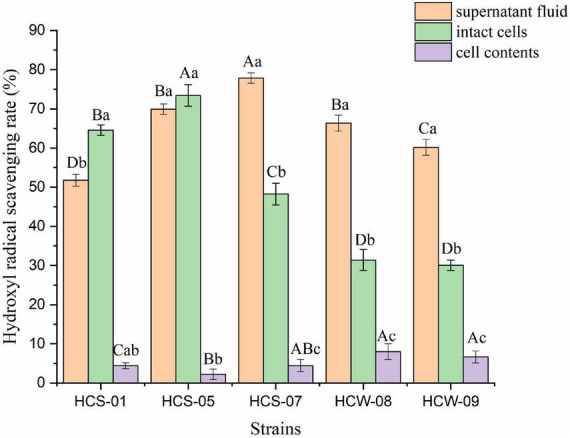
Hydroxyl radical scavenging ability of lactic acid bacteria. Different lowercase letters indicate significant differences among different components of the same strain (*P* < 0.05); different capital letters indicate significant differences among the same components of different strains (*P* < 0.05).

Hydroxyl free radicals have a short survival time, but they oxidize proteins and lipids through electron transfer and hydrogen atom abstraction, which are extremely harmful to organisms. The antioxidant capacity of lactic acid bacteria is positively correlated with the hydroxyl radical scavenging ability (Ahotupa et al., 1996). The hydroxyl radical scavenging ability of different components of lactic acid bacteria is shown in Figure 3. The three components of the five lactic acid bacteria all showed a certain hydroxyl radical scavenging ability, and the scavenging ability of different components of most strains was significantly different. The fermentation supernatants of all strains showed good hydroxyl radical scavenging ability, and the scavenging rate ranged from 51.77 to 77.87%. The three strains HCS-07, HCW-08, and HCW-09 had significantly better ability to clear the fermentation supernatant than the other two components (*P* < 0.05), among which HCS-07 had the highest clearance rate. The complete cell clearance rate of the tested strains ranged from 30.09 to 73.45%, and the complete cell clearance rate of HCS-01 was significantly better than its fermentation supernatant and cell contents (*P* < 0.05); the complete cell clearance rate of strain HCS-05 It was the highest among all tested strains, but there was no significant difference in the ability to clear its fermentation supernatant (*P* > 0.05). The ability of scavenging hydroxyl radicals in the cell contents of all strains was relatively weak, and the scavenging rate ranged from 2.21 to 4.42%. According to the results, the active substances for scavenging hydroxyl radicals of various strains generally exist in the fermentation supernatant of lactic acid bacteria, and the cell surfaces of some strains also have the ability to scavenge hydroxyl radicals, but its activity is weak.

#### Analysis of superoxide anion radical scavenging activity

**FIGURE 4:**
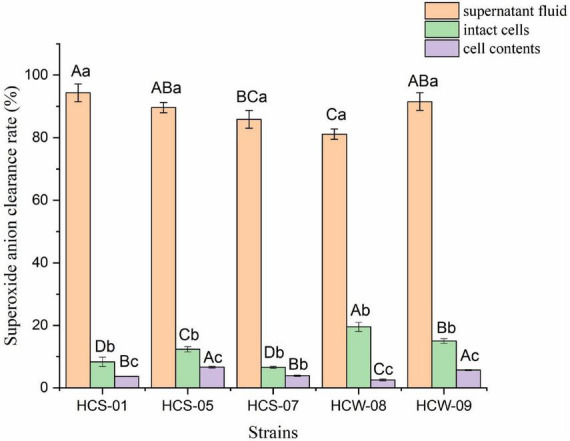
Superoxide anion free radical scavenging ability of lactic acid bacteria. Different lowercase letters indicate significant differences among different components of the same strain (*P* < 0.05); different capital letters indicate significant differences among the same components of different strains (*P* < 0.05).

The direct action of superoxide anion on nucleic acid, protein and other biological macromolecules will cause damage to the cell membrane, and many free radicals in the organism are obtained by the transition of superoxide anion, such as hydroxyl radicals (Virtanen et al., 2007). The hydroxyl radical scavenging ability of different components of lactic acid bacteria is shown in Figure 4. The scavenging ability of superoxide anion radicals in the fermentation supernatant of the same strain was significantly higher than that of the other two components (*P* < 0.05), and the scavenging rate ranged from 81.13 to 94.34%, among which HCS-01 had the highest scavenging rate; The superoxide anion scavenging rate of intact cells of the tested strains was significantly higher than that of cell contents (*P* < 0.05), but the overall scavenging ability was not strong, and the scavenging rate ranged from 6.51 to 19.51%; The range is 2.52–6.61%, and the ability to scavenge superoxide anion is weak. According to the results, it can be speculated that the supernatant of lactic acid bacteria fermentation has high activity of scavenging superoxide anion free radicals, and there are only trace amounts of active substances that can scavenge superoxide anion on the cell surface and in the contents. This indicated that the strain produced active substances that could scavenge superoxide anion during the fermentation process.

### Analysis of antioxidant enzyme activity of lactic acid bacteria

The antioxidant enzyme activities of the tested lactic acid bacteria are shown in Table 2. Superoxide dismutase (SOD) catalyzes superoxide anion to generate hydrogen peroxide, which can be further decomposed into water and oxygen to protect cells from damage (Amanatidou et al., 2001a). The SOD activities in the fermentation supernatants of each strain were significantly higher than those in the intact cells and cell contents (*P* < 0.05), and the enzyme activities ranged from 9.10 to 15.49 U/mL. The strain HCS-05 had higher enzyme activity than other strains; Most strains had no significant difference in SOD activity in intact cells and cell contents (*P* > 0.05), and the enzyme activities ranged from 2.09–3.48 to 1.52–3.54 U/mL, respectively. Among them, Strain HCW-09 had the highest enzymatic activity in intact cells, and strain HCW-08 had the highest enzymatic activity in cell contents.

**TABLE 2:** Enzyme activity of each component of lactic acid bacteria.

Enzyme activity/(U/mL)	Components	Strains				
		
		HCS-01	HCS-05	HCS-07	HCW-08	HCW-09
	Supernatant fluid	9.10 ± 0.33^Ca^	15.49 ± 0.61^Aa^	11.30 ± 0.84^Ba^	9.47 ± 0.41^Ca^	9.78 ± 0.41^Ca^
SOD	Intact cells	2.09 ± 0.17^Cb^	3.33 ± 0.37^Bb^	2.46 ± 0.25^Cb^	3.48 ± 0.18^Bb^	4.15 ± 0.16^Ab^
	Cell contents	2.45 ± 0.28^Bb^	1.52 ± 0.06^Cc^	2.13 ± 0.20^Bb^	3.54 ± 0.34^Ab^	1.54 ± 0.05^Cc^
		–	–	–	–	–
	Supernatant fluid	–	–	–	–	–
CAT	Intact cells	–	–	–	–	–
	Cell contents	1.21 ± 0.28^ABb^	0.86 ± 0.16^AB^	–	1.60 ± 0.61^A^	0.44 ± 0.10^B^
		–	–	–	–	–
	supernatant fluid	–	–	–	0.03 ± 0.01	–
GSH-PX	intact cells	–	–	–	–	–
	cell contents	–	–	–	0.07 ± 0.01	0.08 ± 0.01

Different capital letters indicate significant differences among strains (P < 0.05); different lowercase letters indicate significant differences among different components of the same strain (P < 0.05).

Catalase (CAT) plays an important role in decomposing hydrogen peroxide into water and oxygen in organisms to prevent hydrogen peroxide from causing damage to the body, and also inhibits the further generation of hydrogen peroxide and superoxide anion free radicals (Liochev, 2013). The fermentation supernatants and intact cell bacterial suspensions of the five lactic acid bacteria had no CAT activity, and the cell contents of all tested strains were found to have low CAT activities, ranging from 0.44 to 1.60 U/mL, among which the CAT activity of strain HCW-08 was significantly higher than other strains.

Glutathione peroxidase (GSH-PX) can promote the decomposition of hydrogen peroxide (Kleniewska et al., 2016). GSH-PX activity was not detected in the complete cell suspension of 5 strains of lactic acid bacteria, and its enzyme activity in the fermentation supernatant and the cell content of each strain were also lower, only weak enzymatic activity was detected in the fermentation supernatant of strain HCW-08, HCW-09 and the cellular content of strain HCW-09.

Based on the above results, it is revealed that SOD enzyme was present in the fermentation supernatant, cell surface and cell contents of lactic acid bacteria, and CAT enzyme was weakly present in the cell contents of most lactic acid bacteria and the cell surface of individual lactic acid bacteria, but not in the fermentation supernatant of lactic acid bacteria. Only weak GSH-PX enzyme activity was detected. The results of the analysis indicated that there were significant differences in the activity and localization of antioxidant enzymes among the strains.

### Effects of lactic acid bacteria fermentation on aerobic stability of total mixed ration

#### Analysis of total mixed ration nutrient composition and fermentation quality

The effects of lactic acid bacteria fermentation on the fermentation quality and aerobic stability of silage have been extensively studied before (Yuan et al., 2015; Wang et al., 2020, 2022). The study found that lactic acid bacteria strains with good antioxidant activity can significantly improve the aerobic stability of fermented feeds and prolong the preservation time of feeds (Li et al., 2021).

After 60 days of fermentation, the TMR fermentation quality of each group is shown in Table 3. Group I was treated without lactic acid bacteria, group II was supplemented with heterofermentative lactic acid bacteria strain HCS-05, group III was supplemented with homofermentative lactic acid bacteria strain HCW-08, group IV was supplemented with strain HCS-05 and HCS-08 at 1:1, and group V was supplemented with experiments the lactic acid bacteria strain HDX1 were stored in the room for fermentation.

**TABLE 3:** Nutrient and fermentation quality of total mixed ration (TMR) after 60 days of fermentation.

	Group					
	
	Initial value	I	II	III	VI	V
pH	6.78 ± 0.02^a^	4.51 ± 0.02^b^	4.09 ± 0.07^c^	4.10 ± 0.06^c^	4.05 ± 0.17^c^	4.23 ± 0.09^c^
DM (%FM)	43.10 ± 0.02^a^	38.23 ± 0.68^c^	39.51 ± 1.74^b^	38.44 ± 2.05^c^	39.69 ± 1.83^b^	38.47 ± 1.07^c^
CP (%DM)	15.28 ± 1.57	14.18 ± 2.33	14.86 ± 1.93	15.09 ± 2.05	15.13 ± 2.12	14.73 ± 1.97
WSC (%DM)	16.15 ± 0.89^a^	7.53 ± 0.87^b^	6.59 ± 0.91^d^	6.86 ± 0.45^c^	6.65 ± 0.43^d^	7.05 ± 0.67^c^
AN (%TN)	–	3.32 ± 0.77	3.24 ± 0.23	3.27 ± 0.59	3.16 ± 0.65	3.30 ± 0.43
LAB (lg CFU/g)	4.16 ± 0.15^d^	7.37 ± 0.16^c^	8.16 ± 0.29^b^	8.21 ± 0.21^b^	8.52 ± 0.15^a^	7.93 ± 0.18^b^
Yeast (lg CFU/g)	6.40 ± 0.32^a^	3.21 ± 0.06^b^	3.12 ± 0.15^b^	3.15 ± 0.08^b^	2.98 ± 0.19^b^	3.19 ± 0.15^b^
Aerobic Bacteria (lg CFU/g)	7.21 ± 0.29^a^	3.54 ± 0.18^b^	1.70 ± 0.05^c^	1.74 ± 0.05^c^	1.59 ± 0.06^c^	1.82 ± 0.11^c^
Mold (g CFU/g)	6.11 ± 0.10^a^	1.67 ± 0.58^b^	ND	ND	ND	1.00 ± 0.02^b^

Different lowercase letters indicate significant differences between peer data (P < 0.05), ND, not detected; DM, dry matter; CP, crude protein; WSC, water soluble carbohydrates; AN, ammonia nitrogen.

Compared to pre-fermentation TMR, the pH of each treatment group decreased significantly, and the pH of group II, III, IV, and V inoculated with lactic acid bacteria after 60 days of fermentation was significantly lower than that of group I without lactic acid bacteria (*P* < *0.05*). This indicated that the growth and fermentation of lactic acid bacteria produced a large amount of organic acid, which decreased the pH. There was no significant difference in pH between the lactic acid bacteria treatment groups (*P* > 0.05); the dry matter (DM) and water soluble carbohydrate (WSC) of each treatment group decreased significantly (*P* < 0.05), the WSC decreased significantly, which may be due to the fact that WSC was used as a carbon source by lactic acid bacteria to multiply during the fermentation process, and the degree of decline also reflected the growth of lactic acid bacteria are not obvious. The ammonia nitrogen content of each treatment group was in the range of 3.16–3.32, with no significant difference, and the fermentation quality was good.

After 60 days of fermentation, the number of lactic acid bacteria in each treatment group increased significantly compared with that before fermentation (*P* < 0.05), among which group IV was significantly higher than other treatment groups, and each lactic acid bacteria treatment group was significantly higher than the untreated group (*P* < 0.05); The numbers of yeast and aerobic bacteria in all treatments were significantly lower than the initial value. The number of aerobic bacteria in the lactic acid bacteria-treated group was significantly lower than that in the untreated group (*P* < 0.05), and the difference in the number of yeasts among the groups was not significant (*P* > 0.05). Except for a small amount of molds detected in groups I and V, no molds were detected in other groups, and the number of molds in group V was significantly lower than that in group I.

#### Analysis of aerobic stability of fermented total mixed ration

In this experiment, during the 12 days of FTMR unpacking and aerobic exposure, the effects of different lactic acid bacteria treatments on the aerobic stability of TMR were studied by tracking and monitoring the changes of microorganisms, pH and temperature at each stage. The results are as follows:

##### Microbial changes following fermented total mixed ration aerobic exposure

**FIGURE 5:**
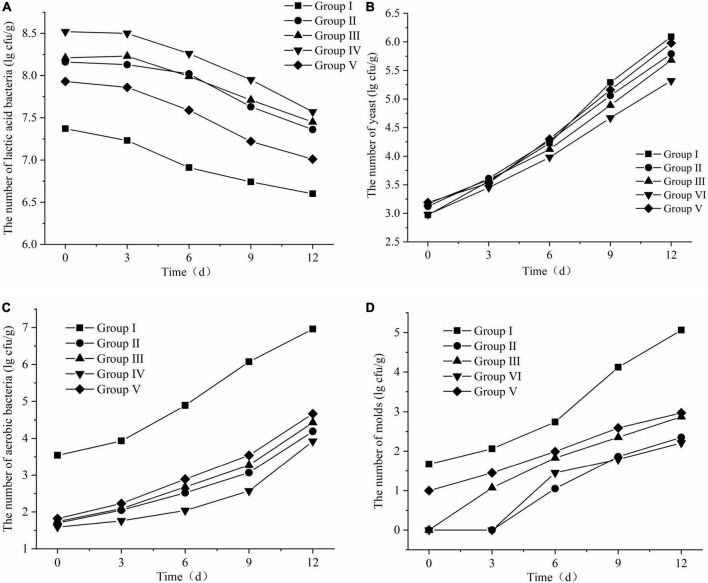
Changes in microbial of fermented total mixed ration (FTMR) during the aerobic period. **(A)** Lactic acid bacteria, **(B)** yeasts, **(C)** aerobic bacteria, and **(D)** molds.

The changes in lactic acid bacteria, yeast, aerobic bacteria and mold during FTMR aerobic exposure are shown in Figure 5. The number of lactic acid bacteria decreased with time of aerobic exposure, while yeast, mold, and aerobic bacteria showed an overall upward trend. As shown in Figure 5A, the number of lactic acid bacteria in groups II, III, and IV did not change significantly on the 3rd day of aerobic exposure, began to decline on the 6th day, and decreased significantly on the 9th and 12th days. The number of lactic acid bacteria in group I without lactic acid bacteria treatment was significantly lower than that in the lactic acid bacteria-treated group, and group V was slightly lower than the other three groups. The order of the final number of lactic acid bacteria from high to low was: group IV > group III > group II > Group V > Group I; as shown in Figure 5B, the changes in the number of yeasts in the 5 groups were consistent during aerobic exposure, and on the 3rd and 6th days, the number of yeasts in each group was slightly Overlapping, the final order was: Group I > Group V > Group II > Group III > Group IV; as shown in Figure 5C, the number of aerobic bacteria in all treatments remained unchanged throughout the aerobic exposure stage. Significant growth, the growth trend became larger from the 6th day, and the final number was sorted from high to low as follows: Group I > Group V > Group III > Group II > Group IV; it can be seen from Figure 5D. The mold growth status of each group in the aerobic exposure stage was found. No mold appeared on the 3rd day in groups II and IV, and mold gradually grew after the aerobic exposure in the other three groups. The growth rate of the group on the 9th day was significantly higher than that of the other groups, and the order of the number of molds from high to low after 12 days of aerobic exposure was: group I > group V > group III > group II > group IV. The reason may be that lactic acid bacteria are part-time anaerobic bacteria, which can also grow well under vacuum anaerobic environment, while the growth of yeast and aerobic bacteria is inhibited in this environment, and the TMR sealed fermentation process, lactic acid bacteria multiply to produce a large number of organic acids to reduce the pH of the environment, which also inhibits the growth of many aerobic bacteria and molds (Liu et al., 2011; Yang et al., 2012).

##### pH changes following fermented total mixed ration aerobic exposure

**FIGURE 6:**
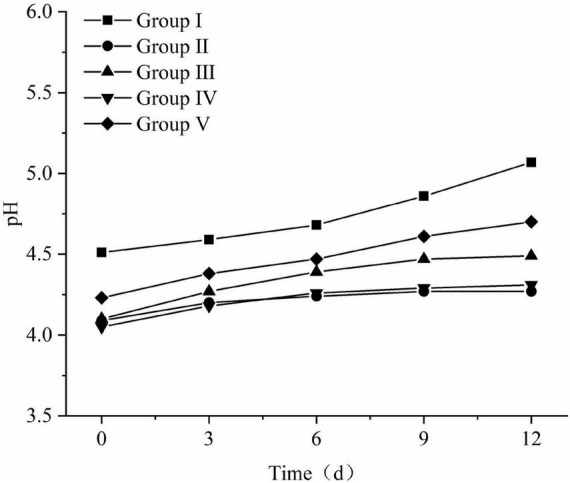
Changes in pH and microbial of fermented total mixed ration (FTMR) during the aerobic period.

The pH changes of FTMR during the aerobic exposure phase are shown in Figure 6. From the 6th day of the aerobic exposure stage, the pH of each treatment group increased significantly. Compared with the other four groups, the pH of the first group increased significantly, and the pH changed rapidly. At the beginning of oxygen exposure, the difference was 0.56; the pH value of group III and group V gradually increased, and the pH value of the 12th day of aerobic exposure was 0.39 and 0.47 at the beginning, 4.49 and 4.70, respectively; the pH of groups II and IV changed slowly, the pH values at the end of aerobic exposure were 4.27 and 4.31, respectively, which were not much different from those at the beginning. The aerobic stability of all groups treated with lactic acid bacteria was significantly better than that of group I without lactic acid bacteria treatment (*P* < 0.05), and the best aerobic stability was group II, followed by group IV and Group III.

By detecting and calculating the difference between the internal temperature and the ambient temperature during aerobic exposure of each treatment, it was found that the stable time of TMR in each group treated with lactic acid bacteria exceeded 228 h, that is, no aerobic spoilage occurred. By comprehensively comparing the microorganisms and pH in the aerobic exposure stage of each treatment group, it can be seen that the lactic acid bacteria treatment has a certain promoting effect on the improvement and improvement of the aerobic stability of FTMR.

The results show that lactic acid bacteria can significantly improve the nutritional quality and fermentation quality of TMR feed, improve its aerobic stability, and delay spoilage. The reason may be that lactic acid bacteria are facultative anaerobic bacteria, which can grow well in a vacuum anaerobic environment, while the growth of yeast and aerobic bacteria is inhibited in this environment, and in the process of TMR sealed fermentation, the reproduction of lactic acid bacteria produces a large of bacteria. Organic acids lower the pH of the environment and also inhibit the growth of many aerobic bacteria and molds. Comparing the fermentation effects of several lactic acid bacteria treatments, it can be found that HCS-05 fermentation and HCS-05 + HCW-08 mixed fermentation have more significant effects on improving the aerobic stability of TMR, which may be due to the fact that HCS-05 is a heterozygous fermentation strain. In addition to a large amount of lactic acid, fermentation products also contain acetic acid and other organic acids. Acetic acid has the effect of inhibiting the growth of yeast (Gomes et al., 2021) and delays the aerobic spoilage of feed. The homofermentative lactic acid bacteria HCW-08 rapidly reproduced and produced a large amount of lactic acid in the early fermentation, and the mixed fermentation with HCS-05 may have a synergistic effect, thereby having a better fermentation effect on TMR feed.

## Conclusion

The results of antioxidant test of lactic acid bacteria showed that all tested strains could tolerate certain concentration of hydrogen peroxide, among which strain HCS-05 and HCS-07 had the best tolerance. The combined free radical scavenging ability of fermentation supernatant of the same strain was generally significantly better than its intact cell suspension and cell content (*P* < 0.05); in terms of antioxidant enzymes of lactic acid bacteria, SOD enzymes were generally present in each of the five lactic acid bacteria In terms of the antioxidant enzymes of *Lactobacillus*, SOD were generally present in all components of the five lactic acid bacteria, while CAT and GSH-PX were weak in all strains. The strains HCS-05 and HCW-08 were selected as the fermenting strains for the silage experiment because of their strong overall antioxidant capacity.

The results of TMR fermentation experiment showed that lactic acid bacteria could significantly improve the nutritional quality and fermentation quality of TMR, improve its aerobic stability and delay spoilage deterioration. Among them, HCS-05 fermentation and HCS-05 + HCW-08 mixed fermentation were more effective in improving the aerobic stability of TMR. The fermentation effect of the screened *Lactobacillus* gastrointestinalis on TMR was better than that of common *Lactobacillus plantarum*, and it has the potential to become a special probiotic for fermented feed for black goats.

## Data availability statement

The raw data supporting the conclusions of this article will be made available by the authors, without undue reservation.

## Author contributions

JY organized the LAB identifications. TY, KT, and RC performed the experiment and the statistical analysis. TY wrote the first draft of the manuscript. All authors contributed to the conception and design of the study, wrote sections of the manuscript, contributed to manuscript revision, read, and approved the submitted version.
